# Predictors of Hospital Stay After Acute Ischemic Stroke in Hospitalized Patients: Retrospective-Cohort Study

**DOI:** 10.1155/crp/7598035

**Published:** 2025-05-05

**Authors:** Zenaw Debasu Addisu, Teshale Ayele Mega

**Affiliations:** ^1^Department of Clinical Pharmacy, College of Medicine and Health Sciences, Bahir University, Bahir Dar, Ethiopia; ^2^Department of Pharmacology and Clinical Pharmacy, School of Pharmacy, College of Health Sciences, Addis Ababa University, Addis Ababa, Ethiopia

**Keywords:** acute ischemic stroke, clinical predictors, Ethiopia, length of hospital stay, stroke

## Abstract

**Background:** The length of hospital stay (LOS) is frequently recognized as an indicator of hospital management efficiency and the quality of care. Patients with acute ischemic stroke (AIS) who experience prolonged LOS are at a higher risk of developing complications such as hospital-acquired infections and gastrointestinal bleeding. These complications can adversely affect clinical outcomes, acting as a primary determinant of poor functional outcomes. However, evidence regarding predictors of the LOS after AIS in Ethiopia is lacking.

**Objective:** Therefore, the objective of this study was to assess clinical predictors of the LOS after AIS among patients admitted to Tibebe Ghion and Felege Hiwot Comprehensive Specialized Hospitals.

**Methods:** A retrospective cohort study was conducted among patients diagnosed with AIS and treated at Tibebe Ghion and Felege Hiwot hospitals from November 2018 to November 2021. Multivariate linear regression analysis was employed to explore predictors of LOS. The slope of regression line (*β*) with its 95% CI is used to declare statistical significance.

**Results:** Of the 278 patients with AIS, 59.7% were male. Stroke-related complications (aspiration pneumonia and urinary tract infections occurred in the hospital in 57 (20.5%), and 12 (4.3%), patients, respectively. The most common neurological deficit observed during hospital admission was limb weakness, affecting 268 patients (96%). The median LOS was 5 days. Among the clinical characteristics, atrial fibrillation (*β* = 7.337, 95% CI: 1.226, 13.448), Limp weakness (*β* = 4.831, 95% CI: 2.330, 7.332), aspiration pneumonia (*β* = 2.089, 95%CI: 1.178, 3.000) and Male sex (*β* = 1.696, 95% CI: 0.851, 2.542), were significant predictors of LOS.

**Conclusion:** In this study, the presence of AF and stroke-related complications, such as aspirational pneumonia, were found to be significant predictors of LOS. Therefore, implementing efficient prevention strategies targeting potentially modifiable risk factors is essential to mitigate the impact of these factors.

## 1. Introduction

The World Health Organization (WHO) defines stroke as “a clinical syndrome of presumed vascular origin, that involves rapidly developing signs of focal or global impairment of cerebral functions lasting more than 24 h or leading to death” [1]. Globally, one in every six individuals will have a stroke in their lifetime, with over 13.7 million having a stroke each year and 5.8 million dying as a result of it [2].

Acute ischemic stroke (AIS), which accounts for approximately 85% of all strokes, is caused by hypoperfusion of brain tissue due to a blockage of blood vessels supplying that specific area of interest, resulting in focal neurologic deficit with or without aphasia, decreased mentation, and respiratory embarrassment [3].

The length of hospital stay (LOS) is frequently recognized as an indicator of efficiency in hospital management as well as in the quality of care [4]. Studies revealed that Patients with AIS who experience prolonged LOS are at a higher risk of developing complications such as hospital-acquired infections and gastrointestinal bleeding [5, 6]. These issues are also linked to an increased likelihood of post-stroke depression and greater disability, both of which can significantly diminish the patient's quality of life [7, 8]. Furthermore, these complications can adversely affect clinical outcomes, acting as a primary determinant of poor functional outcome and leading to unfavorable discharge dispositions in patients with AIS [9, 10]. Moreover, current evidence indicates that patients with a protracted LOS had a 9.3 fold higher chance of unfavorable AIS outcomes at discharge than their counterparts with a shorter LOS [11, 12]. Besides, previous studies have established that LOS is a major cost driver for patients with AIS. Extended hospitalization stays are invariably associated with a rise in medical costs, such as the consumption of beds, medications, care, and health resources [9, 13]. Consequently, therapeutic strategies are developed to shorten hospital stays and to reduce hospitalization costs following a stroke [14]. The results of clinical studies show that AIS can be treated as a medical emergency and that the outcomes can be improved by utilizing the thrombolytic agent, that is recombinant tissue plasminogen activator (rtPA) [15]. Furthermore, studies revealed that short door-to-treatment (DTT) time administration of intravenous tPA (IV tPA) has been correlated to reduced patient LOS, favorable discharge disposition, and more substantial ambulatory status at discharge [16]. Moreover, the American Heart Association recommends that hospitals start IV tPA within 60 min of their arrival [17].

According to the comprehensive review by Feigin et al. the burden of stroke in LMICs is extensive and increasing yet, few patients have been thrombolyzed [9]. In developing nations, acute stroke treatment is primarily symptomatic; thrombolytic and neuroprotective medications are the exception rather than the rule [18].

The duration of hospitalization for acute stroke varies, ranging from one to 2 weeks on average [19, 20]. Longer stays are more likely in older patients and those with more severe strokes, anterior circulation infarctions, and atrial fibrillation [19] As a result, identifying clinical predictors of LOS is crucial for reducing the burden of AIS, and it is useful for planning and organizing AIS treatments in resource-limited countries.

A rising incidence of stroke in Ethiopia is a threat to the healthcare system. Furthermore, financial resources for stroke care and rehabilitation are limited [21, 22]. Despite this, data are scarce in Africa, particularly in Ethiopia, identifying clinical predictors of LOS in patients with AIS. Therefore, this study aimed to identify clinical predictors of LOS after AIS among patients admitted to Tibebe Ghion Specialized Hospital (TGSH) and Felege Hiwot Comprehensive Specialized Hospital (FHCSH). A more thorough awareness of the factors that affect LOS allows us to focus our resources on at-risk patients and enables AIS care units to develop more effective treatment approaches to shorten hospital stays and control medical costs.

## 2. Materials and Methods

### 2.1. Study Design and Setting

A retrospective cohort study was employed at TGSH and FHCSH, two specialized referral public hospitals in Bahir Dar city, North-Western Ethiopia from November 2018 up to November 2021. Bahir Dar City is located 587 km northwest of Addis Ababa, which is the capital city of Ethiopia. The study was based on a similar study design and setting conducted in the tertiary Care Hospitals found in Bahir Dar city using the medical records of AIS patients with atrial fibrillation during the same study period [23]. However, the current study involved a different study population with different study objectives and a different sample size.

### 2.2. Study Population and Patient Enrollment

**Figure 1:**
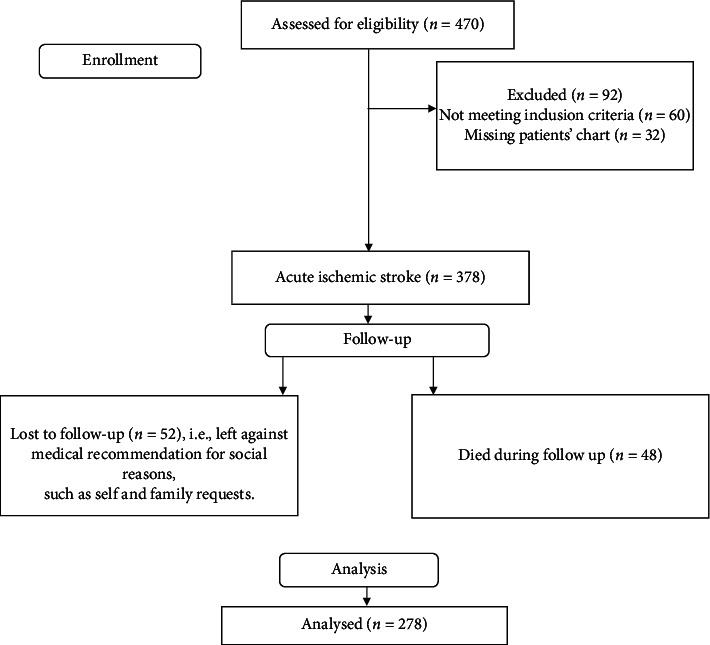
Flowchart showing selection of patients diagnosed with acute ischemic stroke from FHCSH and TGSH.

The study population encompassed all patients with AIS admitted to the medical departments of TGSH and FHCSH between November 2018 and November 2021, who were 18 years old and diagnosed with AIS. Patients with missing medical records, patients with insufficient medical records, patients with CT or MRI-confirmed cerebral hemorrhage, and patients with transient ischemic attack (TIA) were excluded. From 470 eligible patients' charts, 192 patients' charts were removed for a variety of reasons. As a result, the final study included 278 patients (Figure 1).

### 2.3. Data Collection Tool and Study Variables

Using an English version of the checklist, the records of AIS patients admitted to TGCS and FHCS hospitals over 3 years were reviewed. The checklist was developed after reviewing pertinent literature. The specially trained data collectors; two nurses and two pharmacists extracted all relevant data from patient medical records, which included socio-demographics, risk factors, and stroke-related complications during hospitalization. Furthermore, the admission date, discharge date, and LOS were extracted from the patient records using a structured data extraction form.

### 2.4. Outcome Definition and Measurement

The LOS of AIS patients were considered the outcome variable, and as we aimed to determine the key predictors of LOS based on the available admission data, we specifically assessed factors that were measurable at the time of admission. We studied the effect of variables on hospitalization in a multiple-regression model.

### 2.5. Data Analysis

Data analyses were performed using the Statistical Package for Social Sciences (SPSS) version 25. Descriptive analysis was performed and results were presented in text, tables, and charts. Continuous variables were reported using mean, median, and interquartile ranges. Bivariate and multivariate linear regressions were also carried out to assess independent predictors of LOS. Bivariate linear regression was performed to identify candidate variables for multivariable linear regression. Variables with a *p* value ≤ 0.2 in bivariate regression were candidates for multivariable regression. Multivariable linear regression was performed using the backward method to identify independent predictors. Regression coefficients and their 95% confidence intervals together with *p* value < 0.05 were used to identify independent predictors. In order to address possible problems with collinearity, the tolerance and the variance inflation factor (VIF) values of each independent variable were assessed.

### 2.6. Operational Definition of Terms

• AIS: is ischemic stroke with symptom onset-to-arrival time within 24 h to 7 days [24].
• LOS: was defined as the amount of time between hospital admission and discharge. Admission date was defined as the date the patient was admitted to the hospital with AIS or if the patient was already in the hospital for another condition, the day the stroke actually occurred. The date that a patient is transferred from the stroke unit to their home or a rehabilitation facility is referred to as the discharge date.

## 3. Results

Between November 2018 and November 2021, 470 patients with ischemic stroke were enrolled at two hospitals in the region. Of the 470 patient charts assessed for eligibility, 192 patients' charts were eliminated for a variety of reasons. Therefore 278 patients were included in the final analysis Figure 1.

The majority of patients 167 (60%) were from FHCSH and 111 (40%) were from TGSH.

### 3.1. Socio-Demographic Characteristics of the Patients

Of the 278 eligible patients' charts, more than half, 166 (59.7%) of the patients were male and 112 (65.5%) were rural residents. Besides 201 (72.3%) were married while 60 (21.6%) were widowed. The majority, 147 (52.9%) of patients were farmers followed by 40 (14.4%) unemployed. Regarding religion, 231 (83.1) were Orthodox Christians. Of the total patients, 191 (68.7%) had no formal education (Table 1).

### 3.2. Baseline Characteristics of the Study Participants

Of the 278 patients admitted with AIS, 69 (24.8%) were diagnosed with AF.  Table 2 demonstrates the baseline characteristics of AIS patients during hospital admission. As for time from symptom onset to hospitalization, only 52 (18.8%) patients were hospitalized within 3 h of symptom onset. Regarding comorbidity, 53 (19.1%) patients with AIS had valvular heart disease (i.e., CRVHD, prosthetic heart valve, mechanical heart valve), 17 (6.1%) had coronary artery disease (CAD), and 10 (3.6%) patients had dilated cardiomyopathy (DCMP). Most patients, that is, 137 (71.4%), patients had low-density lipoprotein levels greater than 100 mg/dL and had hyperlipidemia at hospital admission. Regarding patients' hypertension status, 120 (43.2%) patients were known Hypertensive patients while 39 (14%) patients were newly diagnosed hypertensive patients. Similarly, on the basis of the diabetes status of the patient, 259 (93.2%) patients had normal blood glucose levels whereas 8 (2.9%), and 11 (4%) were newly diagnosed diabetes and known diabetes patients respectively.

Moreover, 152 (58.5%) never drink alcohol, 78 (30.1%) were current alcohol users and only 29 (11.2%) patients were former alcohol users. Similarly, most patients never smoked cigarettes, 5 (2%) patients were former smokers who had quit smoking before 1 year, and 16 (6.5%) were current smokers.

#### 3.2.1. Neurologic Deficits Observed During the Admission of the Study Participants

The most common neurological deficit observed during hospital admission was limb weakness, affecting 268 patients (96%), followed by aphasia in 249 cases (90%), facial palsy in 144 (52%), and loss of consciousness in 116 (42.0%). Conversely, loss of memory, loss of vision, and bilateral hearing loss were less frequently observed at the time of admission. These results in neurologic deficits observed during hospital admission, presented in Supporting Information Table S1.

#### 3.2.2. Complications During Hospitalization in the Study Participants

Regarding with in hospital stroke related complication 57 (20.5%) and 12 (4.3%) patients were acquired aspirational pneumonia and urinary tract infection respectively. 18 (6.4%) patients developed hypokalemia, while 9 (3.2%) patients experienced hemorrhagic transformation.  Table 3 shows complications that occur in hospitals.

### 3.3. Predictors for LOS

The LOS was assessed from the day the patient was admitted to the hospital with AIS or, if the patient was already hospitalized for another condition, from the day of the actual stroke to the day the patient was transferred from the stroke unit to home, or a rehabilitation facility. The median LOS was 5 days, ranging from 1 to 18 days.

A linear regression analysis was performed to assess the predictors of the LOS. Variables with *p* < 0.2 level in the bivariate linear regression model; the presence of atrial fibrillation, aspirational pneumonia, limp weakness, acute gastrointestinal hemorrhage, the presence of valvular heart disease, Male sex, and newly diagnosed Diabetes mellitus, were further treated multivariate linear regression analysis to identify independent predictors of LOS (Table 4). Among these four variables were identified as correlated with LOS by backward multivariate linear regression methods.

For the current model, multicollinearity problems were not present in the models because all tolerance levels were higher than 0.2 and the VIF values for all independent variables were < 10. Since the data in all of the models' standardized residuals were normally distributed, the requirements of the linear regression model were also fulfilled (*p* < 0.05).

Hence, the results of the multivariate linear regression analysis showed that the presence of atrial fibrillation was associated with an increase, in LOS by 7.337 times (*β* = 7.337, 95% CI: (1.226–13.448)). Furthermore, patients who acquired aspiration pneumonia during hospitalization a 2.089 fold (*β* = 2.089, 95% CI: (1.178–3.000)) increase in LOS.

In addition, patients with male sex associate increased LOS by 1.696 times (*β* = 1.696, 95% CI: (0.851–2.542)). Besides, patients with limp weakness associated with, increased LOS by 4.831 times (*β* = 4.831, 95% CI: (2.330–7.332)).

## 4. Discussion

When managing acute stroke patients in hospitals, the LOS is the primary factor that influences costs [25]. Furthermore, a better understanding of the factors that affect LOS allows us to focus our resources on high-risk patients and enables AIS units to develop more effective treatment approaches to reduce hospital length of stay and medical expenses. The analysis of the LOS can give significant data for planning and policy in the healthcare system.

This study assesses the clinical predictors of the LOS for patients admitted with AIS and who survived and were discharged from acute ischemic episodes. The median LOS was 5 days, ranging from 1 to 18 days. The multiple regression analysis showed that the presence of AF, Limp weakness as well as stroke-related comorbidities such as aspiration pneumonia and male sex, were significant predictors of the LOS.

Research conducted in Western nations has revealed a significant link between male gender and extended hospital stays [26]. Our study corroborates these findings among AIS patients, showing that being male is associated with longer LOS. It remains uncertain whether the influence of gender on LOS is indicative of cultural disparities or stems from other factors.

The current study also demonstrated that patients who acquired aspiration pneumonia during hospitalization had a 2.089-fold (*β* = 2.089, 95% CI: (1.178–3.000)) increase in LOS. It considerably extended hospital stays, as compared with those patients who did not acquire aspiration pneumonia during their hospital stay. Similar to this finding study in Spain, Barcelona [27], reported that stroke-associated infection, particularly aspirational pneumonia, was independently associated with AIS and prolonged LOS (AOR = 1.78 (1.37, 2.33) *p* < 0.001). Moreover, according to studies reports neurogenic dysphagia affects 22%–49% of acute stroke patients [28]. It is a high-risk factor for a variety of sequelae, particularly aspiration pneumonia, studies recommend that early swallow screening reduces the incidence of aspiration pneumonia and the length of stay in hospitals [28–30].

The current study also reported that in the presence of AF, there was a 7.337-fold (*β* = 7.337, 95% CI: (1.226–13.448)) increase in LOS. Previous studies had also reported that patients with AIS and AF had a longer hospital stay [31–33]. A longer duration of stay in patients with AF may be owing to a higher risk of in-hospital medical complications, which would necessitate further treatment before the patient is discharged.

Clinical indicators associated with stroke severity, such as limb weakness, degree of paralysis, and unilateral neglect as assessed by the NIHSS, have the most significant influence on LOS [27, 34]. In the current study, it was found that patients with limb weakness during their hospitalization experienced a 4.831 fold increase in the LOS (*β* = 4.831, 95% CI: (2.330–7.332)). This may be attributed to the fact that patients with limb weakness are typically slow to ambulate and unable to care for themselves because of their significant neurological impairments, leading to reduced functional independence in everyday tasks, which consequently can prolong the length of hospitalization [35].

### 4.1. Strengths and Limitations of This Study

It is the sole cohort study in Ethiopia assessing clinical predictors of LOS in patients with AIS. However, the study has limitations, including the retrospective collection of clinical factors, which is susceptible to recall bias. Additionally, many patient charts, including those of deceased Patients who were lost to follow-up, were excluded which may introduce bias. Furthermore, our analysis was hindered by missing crucial variables in patient records, such as the etiology of AIS, potentially affecting LOS.

## 5. Conclusion and Implication of the Study

In this study, the presence of atrial fibrillation, as well as stroke-related complications such as aspiration pneumonia were significant predictors of LOS. Hence implementing efficient prevention strategies targeting potentially modifiable risk factors is essential to mitigate the impact of these factors. As this study was conducted in a small number of hospitals, additional prospective investigation is required. Future research should look into the impact of various potential predictors of LOS, such as the etiology of AIS.

## Figures and Tables

**Table 1:** Socio-demographic characteristics of patients diagnosed with acute ischemic stroke from two hospitals from November 2018 to November 2021 (*n* = 278).

Socio-demographic characteristic		Total (278)
Sex	Male	166 (59.7%)
	Female	112 (40.3%)

Age	≤ 53	60 (21.6%)
	54–64	55 (19.8%)
	65–74	87 (31.3%)
	75–84	62 (22.3%)
	≥ 85	14 (5%)

Marital status	Single	9 (3.2%)
	Married	201 (72.3%)
	Divorced	8 (2.9%)
	Widowed	60 (21.6%)

Residency	Urban	96 (34.5%)
	Rural	182 (65.5%)

Religion	Orthodox	231 (83.1%)
	Protestant	25 (9%)
	Muslim	20 (7.2%)
	Catholic	2 (0.7%)

Educational status	No formal education	191 (68.7%)
	Primary school (1–8)	28 (10.1%)
	Secondary school (9–12)	26 (9.4%)
	Collage/university	33 (11.9%)

Employment level	Employed	29 (10.4%)
	Farmer	147 (52.9%)
	Unemployed	40 (14.4%)
	Housewife	36 (12.9%)
	Self-employed	26 (9.4%)

**Table 2:** Baseline characteristics of acute ischemic stroke patients (*n* = 278).

Variables	Category		Total (%)
Time from symptom onset to hospital	≤ 3 h		52 (18.8%)
	> 3 h		225 (81.2%)

LDL level	LDL < 100		55 (28.6%)
	LDL ≥ 100 mg/dL		137 (71.4%)

Hypertensive status	Normal blood pressure		119 (42.8%)
	Newly diagnosed HTN		39 (14%)
	Known hypertensive patient		120 (43.2%)

Diabetes mellitus status	Normal blood glucose level		259 (93.2%)
	Newly diagnosed DM		8 (2.9%)
	Known DM patient		11 (4%)

Alcohol	Never drink alcohol		152 (58.5%)
	Current alcohol use		78 (30.1%)
	Former drinker		29 (11.2%)

Smoking	Never smoke		227 (91.5%)
	Current smoker		16 (6.5%)
	Former smoker		5 (2%)

GCS	Mild (13–15)		159 (57.2%)
	Moderate (9–12)		58 (20.9%)
	Severe (< 8)		61 (21.9%)

Comorbidity	VHD	Present	53 (19.1%)
		Absent	225 (80.9%)
	HHD	Present	22 (7.9%)
		Absent	256 (92.1%)
	CHD	Present	17 (6.1%)
		Absent	261 (93.9%)
	DCMP	Present	10 (3.6%)
		Absent	268 (96.4%)
	AF	Present	69 (24.8%)
		Absent	209 (75.2%)

Abbreviations: AF, atrial fibrillation; AIS, acute ischemic stroke; CHD, coronary heart disease; DCMP, dilated cardiomyopathy; GCS, glasco coma scale; HHD, hypertensive heart disease; LDL, low-density lipoprotein; VHD, valvular heart disease.

**Table 3:** Stroke-related complications during hospital stay in a patient with acute ischemic stroke.

Variables	Total (%)
Aspirational pneumonia	57 (20.5%)
Urinary tract infection	12 (4.3%)
Urinary incontinence	5 (2.6%)
Acute renal failure	5 (1.8%)
Hypokalemia	18 (6.4%)
Acute gastrointestinal hemorrhage	3 (1.1%)
Hemorrhagic transformation	9 (3.2%)
Pulmonary embolism	1 (0.4)
Post-stroke depression	6 (2.1%)
Increase intracranial pressure	4 (1.4%)
Hospital-acquired pneumonia	6 (2.1)
Deep venous thrombosis	1 (0.4%)
Bedsore	4 (1.4)

**Table 4:** Independent predictors of length of hospital stay in patients with an acute ischemic stroke.

Variables	Category	Unadjusted slope (*β*) of regression line		Adjusted slope (*β*) of regression line	
		*β* (95% CI)	*p* value	*β* (95% CI)	*p* value
Newly diagnose diabetes mellitus	No	Reference			
	Yes	1.875 (−0.516, 4.266)	0.124		

Atrial fibrillation	No	Reference			
	Yes	8.447 (1.847, 15.106)	0.012	7.337 (1.226, 13.448)	0.019⁣^∗^

Aspirational pneumonia	No	Reference			
	Yes	2.481 (1.531, 3.431)	≤ 0.001	2.089 (1.178, 3.000)	≤ 0.001⁣^∗^

Acute gastrointestinal hemorrhage	No	Reference			
	Yes	2.810 (−1.061, 6.681)	0.154		

Sex	Female	Reference			
	Male	1.904 (1.003, 2.806)	≤ 0.001	1.696 (0.851, 2.542)	≤ 0.001⁣^∗^

Valvular heart disease	No	Reference			
	Yes	1.554 (0.548, 2.559)	0.003		

Limp weakness	No	Reference			
	Yes	5.225 (2.533, 7.918)	≤ 0.001	4.831 (2.330, 7.332)	≤ 0.001⁣^∗^

⁣^∗^The Statistically significant predictor of length of hospital stays with *p* < 0.05 in multivariate linear regression analysis using the backward method.

## Data Availability

The data that support the findings of this study are available from the corresponding author upon reasonable request.

## Nomenclature

AF: Atrial fibrillation
AIS: Acute ischemic stroke
LOS: Length of hospital stay
NIHSS: National Institutes of Health Stroke Scale
WHO: World Health Organization

## Supporting Information

The supporting information files includes Table S1 as mentioned in the text (supporting information Table S1).
