# Supplementary material for: Predictors of Hospital Stay After Acute Ischemic Stroke in Hospitalized Patients: Retrospective-Cohort Study
Source: Cardiol Res Pract. 2025 May 5;2025:7598035. doi: 10.1155/crp/7598035 (PMC12069843; doi:10.1155/crp/7598035)
Supplement: Supporting Information — Additional supporting information can be found online in the Supporting Information section. [file 7598035.f1.docx]

Supplementary Table S1:Neurologic deficits during Hospital admission

| Neurologic deficits during admission | Total (%) |
| --- | --- |
| LOC | 116 (42.0%) |
| Aphasia | 249 (90%) |
| facial palsy | 144 (52 %) |
| Limp Weakness | 268 (96%) |
| Urinary incontinence | 68 (24 %) |
| Headache | 118 (42. %) |
| Convulsions/ABM | 24 (8.6%) |
| Swallowing difficulty/dysphagia | 5 (1.8%) |
| loss of memory | 1(0.4%0 |
| Loss of vision | 2(0.7%) |
| Bilateral hearing loss | 1(0.4%) |
| Neck stiffness | 1(0.4%) |
